# How to measure the pulse

**Published:** 2013

**Authors:** Dianne Pickering

**Affiliations:** Former Nurse Advisor, Community Eye Health Journal

**Figure:**
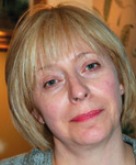
Dianne Pickering

Before surgery, eye patients must be assessed for their suitability for surgery. Taking the pulse allows us to find out what the patient's heart rate is and to assess the strength, regularity, and character of the pulse. Irregularities might indicate a heart problem and must be investigated.

Taking the pulse also provides an initial recording (a ‘baseline’) that will enable us to compare future measurements and monitor changes in our patient's condition.

The pulse can be measured at several points in the body. These points are where an artery is situated just under the skin, where it can be compressed against a bone, allowing us to feel each beat.

This article will cover the measurement of the pulse at the radial point (inside the wrist, see Figure 1) as this is the most common point at which to measure the pulse of eye patients.

**NOTE:** Many things-such as anxiety, pain and fever-can raise the patient's pulse (heart rate) and certain medications such as beta blockers or digoxin can lower it; all of these reasons should be considered when assessing and recording the patient's pulse. If you are taking repeat measurements of the same patient, try to measure the pulse under the same conditions each time.

## What is normal?

A normal pulse is regular and strong. Heart rates, and therefore pulse rates (number of beats per minute) change with age and can vary between individuals of the same age.

**Table 1.** Normal pulse rate range, by age

Age	Pulse rate (beats per minute)
Newborn (resting)	100-180
Infant (resting)	80-150
Child 26 years	75-120
Child 6-12 years	70-110
Adolescent-adult	60-90

## You will need

- A watch that has a second hand
- A chart to record the pulse measurement
- A black pen.

## Before you begin

1. Wash your hands-this will help to prevent cross-infection.
2. Explain what you are about to do. This will help the patient to understand what is about to happen and will make it easier for them to co-operate.

**Figure 1:**
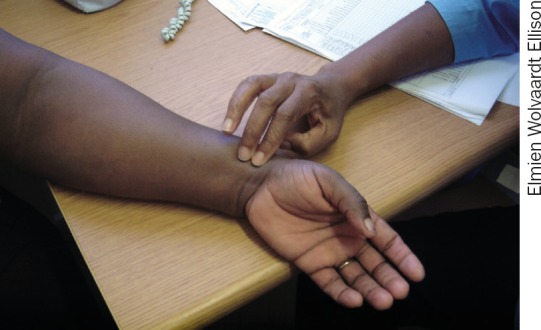


## Procedure

1. Ask whether the patient has walked, climbed stairs, or otherwise exerted themself in the last 20 minutes. If not, you can proceed. If the answer is yes, wait 20 minutes before taking the reading. This will help to prevent false readings.
2. Make sure the patient is relaxed and comfortable.
3. Place the tips of your first and second finger on the inside of the patient's wrist (Figure 1).
4. Press gently against the pulse. Take your time to note any irregularities in strength or rhythm.
5. If the pulse is regular and strong, measure the pulse for 30 seconds. Double the number to give the beats per minute (e.g.: 32 beats in 30 seconds means the pulse is 64 beats per minute). If you noticed changes in rhythm or strength, you must measure the pulse for a full minute.
6. Record the pulse rate (the number of beats per minute) in the patient's notes and describe its strength and rhythm. Compare the pulse rate with the values in the Table land record whether the pulse is normal, sloworfast. Any abnormalities should be recorded and reported to the senior nurse and doctor.
7. Strength of the pulse isa very subjective measurement, but an experienced nurse will compare it with what has been felt previously in other patients. Describe the puse as ‘weak’, ‘faint’, ‘strong'or'bounding’.
8. Think about the rhythm of the pulse. Is it regular? If irregular, in what way? Cardiac problems may present as a regular missed beat, for example, so is the irregularity regular (described as regularly irregular) or is there no pattern (described as irregularly irregular)?
9. Discuss with your patient the result of the pulse measurement and if any further investigations are required.
10. Wash and dry your hands.

### Sources

Nursing and midwifery: a practical approach. Sally Huband, Pam Hamilton Brown and Gillian Barber Macmillan Education Royal Marsden Hospital Manual of Clinical Nursing Procedures **www.clinicalskills.net**

